# Case Report: Preoperative treatment of portal hypertension by splenic artery embolization for safe major hepatectomy: experience in three patients

**DOI:** 10.3389/fsurg.2026.1674944

**Published:** 2026-03-25

**Authors:** Duygu Gürel, Tevfik Güzelbey, Oğuzhan Aydın, Serhat Kaya, Özgür Bostancı, İlgin Özden

**Affiliations:** 1 Liver Transplantation & Hepatopancreatobiliary Surgery Unit, Department of General Surgery, Başakşehir Çam & Sakura City Hospital, İstanbul, Türkiye; 2 Department of Pediatric Surgery and Pediatric Urology, Prof. Dr. Cemil Taşcıoğlu City Hospital, İstanbul, Türkiye; 3 Department of Radiology, Başakşehir Çam & Sakura City Hospital, İstanbul, Türkiye; 4 Hepatopancreatobiliary Surgery Unit, Department of General Surgery, Acıbadem Maslak Hospital, İstanbul, Türkiye

**Keywords:** case report, cholangiocarcinoma, colorectal liver metastasis, portal hypertension, post-hepatectomy liver failure, splenic artery embolization

## Abstract

Post-resection portal pressure is one of the most important determinants of post-hepatectomy liver failure. Therefore, preexisting portal hypertension is a significant risk factor for PHLF. Preoperative splenic artery embolization (SAE) was used to alleviate portal hypertension before right-sided hepatectomy in three non-cirrhotic female patients with colorectal liver metastases (45), perihilar cholangiocarcinoma (55), and intrahepatic cholangiocarcinoma (69). Two had received chemotherapy and one had undergone radiotherapy, transarterial radioembolization as well. All had thrombocytopenia (79 × 10^3^/µL, 51 × 10^3^/µL, 58 × 10^3^/µL respectively), two patients had splenomegaly and one had esophageal varices. Partial SAE was performed in two cases (upper pole preserved) and total SAE in one with coils and plug. This resulted in normalization of platelet counts within two weeks (419 × 10^3^/µL, 340 × 10^3^/µL, 159 × 10^3^/µL respectively) and regression of varices. The calculated future remnant liver volumes were 47%, 63% and 62% respectively. All subsequently underwent surgery: extended right hepatectomy including the middle hepatic vein (H5678-MHV), right hepatectomy-total caudate lobectomy-Roux-Y cholangiojejunostomy (H15678-B) and right hepatectomy (H5678). The perihilar cholangiocarcinoma patient developed grade B post-hepatectomy liver failure and recovered with supportive treatment. All patients were discharged. One patient died of recurrent disease at 17 months, whereas the remaining two are disease-free at 21 and 36 months. SAE should be considered for portal flow modulation in major hepatectomy candidates with portal hypertension.

## Introduction

Post-resection portal vein pressure is one of the most important determinants of post-hepatectomy liver failure (PHLF) (1, 2). Therefore, preexisting portal hypertension is a significant risk factor in major hepatectomy candidates (3–5). For hepatocellular carcinoma in the setting of chronic liver disease, this problem is circumvented by offering liver transplantation instead of liver resection in appropriate cases (3, 6). A less common, but successful strategy at some very experienced centers is to alleviate portal hypertension by splenectomy in patients with preserved liver function (3, 7). This concept -portal flow modulation- may be applied in portal hypertensive patients with liver metastases and cholangiocarcinomas. For example, successful use of splenic artery ligation (SAL) in four cases with colorectal liver metastases and chemotherapy-induced liver injury was reported by Schwarz et al. in 2014 (8). In 2022, Theodoraki et al. communicated favorable experience with SAL in 13 patients, 7 of whom had metastatic tumors (9). Junrungsee et al. have shown that SAL effectively reduces the risk of clinically significant PHLF in patients with elevated portal venous pressure following hepatectomy in a randomized controlled trial (5).

The radiological counterpart to splenic artery ligation is splenic artery embolization (SAE) (10, 11). Beneficial results have been reported in the treatment of chemotherapy-associated thrombocytopenia caused by hypersplenism resulting from chemotherapy-induced liver injury, extensive liver metastases and cirrhosis (12–14). It has been applied preoperatively to prevent and postoperatively to treat the small for size syndrome caused by portal hyperperfusion in the setting of living donor transplantation (11, 15). SAE offers several practical advantages over surgical splenectomy or splenic artery ligation: it is minimally invasive and repeatable; although they are rare, should procedure-related complications occur (10, 11), they can can be treated preoperatively. By reducing splenic inflow, SAE effectively alleviates portal hypertension, and potentially improves postoperative liver function and surgical outcomes.

Here, we present the favorable outcomes of three noncirrhotic patients (one patient each with colorectal metastases, perihilar cholangiocarcinoma and intrahepatic cholangiocarcinoma) in whom we performed SAE to alleviate portal hypertension before hepatectomy.

## Case descriptions

### Case 1

A 45-year-old female patient with rectosigmoid adenocarcinoma had two liver metastases (the larger one was 111 × 76 mm and located in the right lobe). She initially underwent a semi-urgent low anterior resection, para-aortic lymph node dissection, total abdominal hysterectomy and bilateral salpingo-oophorectomy by the colorectal surgery team due to closed perforation and adnexial invasion of the rectosigmoid tumor. She received oxaliplatin-based chemotherapy for five months after colorectal surgery: regression was observed in both metastases (the larger one regressed to 73 × 55 mm), PET-CT imaging revealed no evidence of extrahepatic disease. The patient was referred for hepatectomy. She had no history of radiotherapy or previous hepatic interventions prior to hepatectomy.

**Figure 1:**
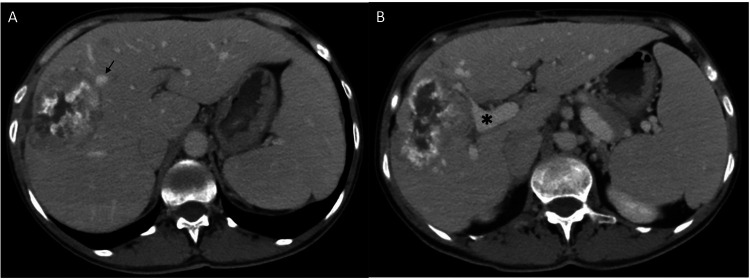
**(A,B)** The relationship between the larger of the two metastases with the middle hepatic vein (arrow) and major tributaries of the right portal vein branch (*).

**Figure 2:**
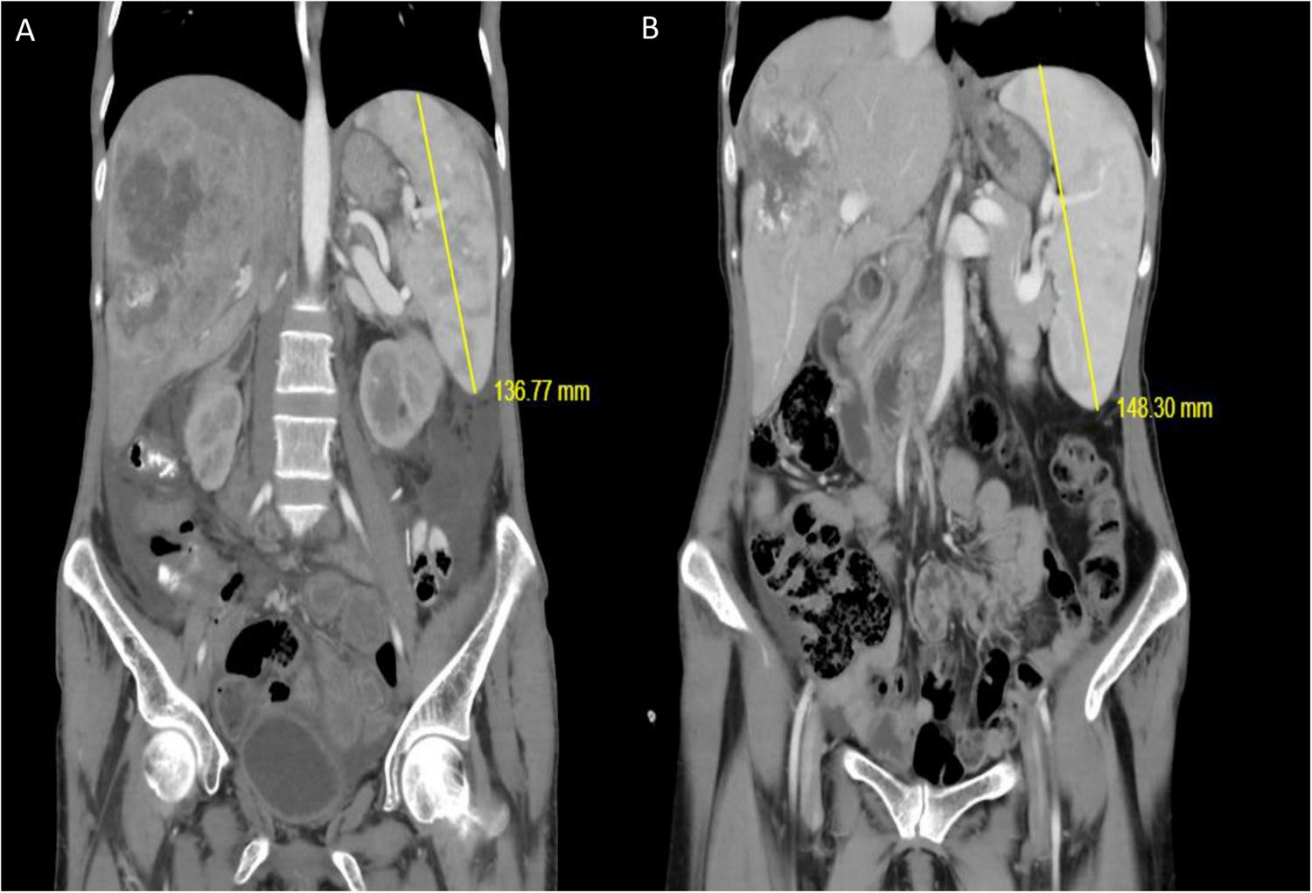
**(A,B)** Development of splenic enlargement in the course of hepatectomy; the two films are 4 months apart.

The association of the larger of the metastases with the vascular structures (Figures 1A,B), required an extended right hepatectomy for an R0 resection. The calculated future remnant liver volume was 47% (16). However, examination of the available images revealed splenic enlargement accompanied by progressive thrombocytopenia (down from 316 to 378 × 10^3^/µL to 79-123 × 10^3^/µL) (Figures 2A,B). Although there were no varices on upper gastrointestinal endoscopy, portal hypertension due to chemotherapy was considered. To minimize postoperative complications, partial splenic artery coil embolization was performed by preserving the arterial circulation of the upper pole. The C-reactive protein (CRP) level gradually increased from a normal level to 133 mg/L on the fourth day post-embolization and then gradually decreased back to 5 mg/L (0–5). Concurrently, the patient's platelet count reached 419 × 10^3^/µL. Extended right hepatectomy including the middle hepatic vein (H5678-MHV) (17) and cholecystectomy were performed on the 13th day post-embolization. She had an uneventful postoperative course with no remarkable ascites and a maximum total bilirubin level of 2.6 mg/dL and an INR of 1.6, both of which gradually returned to normal. PHLF was not observed (18). She was discharged on the 8th postoperative day. She has had no recurrence during 3 years of follow-up.

### Case 2

**Figure 3:**
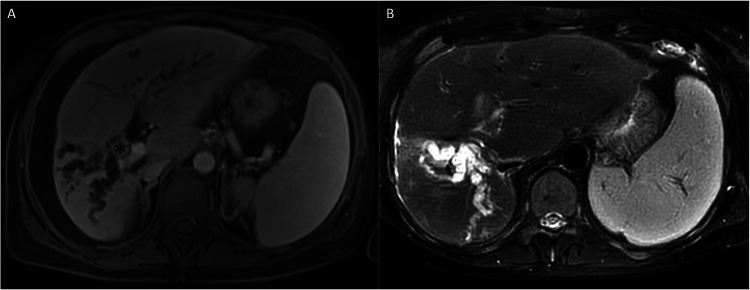
**(A)** Intraductal mass lesion (asterix) on contrast-enhanced T1 axial MRI series. **(B)** T2- weighted MRI image after PTBD to the left lobe and ascites resolution.

A 55-year-old female patient with a history of epilepsy and hyperthyroidism underwent various inconclusive investigations for cholestasis (total bilirubin 2.2 mg/dL, direct bilirubin 2 mg/dL, GGT 523 U/L) over a period of three months at other centers. During this interval, her platelet count decreased from 142 to 194 × 10^3^/µL to 80 × 10^3^/µL, she developed ascites and splenomegaly (Figure 3A); percutaneous external biliary drainage (PTBD) was performed. Ascites had resolved at the time of admission, and there was no history of prior radiotherapy or chemotherapy before referral to our center.

The available radiological examination suggested the possibility of a slow-growing perihilar cholangiocarcinoma that required a right hepatectomy and caudate lobectomy (Figure 3A). However, grade 1 esophageal varices and an antral ulcer were observed on upper gastrointestinal endoscopy at our institution. Medical treatment for peptic ulcer was started. The patient's platelet level, which was 80 × 10^3^/µL in the beginning of hospitalization, dropped to 51 × 10^3^/µL in the course of cardiovascular, neurologic and endocrine work up. Although the right lobe had become atrophic (the calculated future remnant liver volume: 63%) (16) (Figure 3B), the available findings precluded a major hepatobiliary resection.

Partial splenic artery coil embolization to the middle and lower parts of the spleen was performed. Starting from the 4th day after embolization, the platelet count gradually increased and reached 340 × 10^3^/µL in the second week; the CRP levels increased to 200 mg/L on the third day and gradually decreased to 21 mg/L in the second week. The eventual biochemical data were as follows: albumin 35 g/L, INR 1.2, GGT 254 U/L, total bilirubin 0.7 mg/dL, direct bilirubin 0.5 mg/dL. Follow up upper gastrointestinal endoscopy revealed healing of the ulcer and disappearance of the varices. Subsequently, the patient underwent right hepatectomy- total caudate lobectomy- Roux-Y cholangiojejunostomy (H15678-B) (17) in the third week post-embolization. The maximum postoperative INR level was 1.9 and the total bilirubin level was 2 mg/dL, both gradually decreasing to within normal range at discharge. However, PHLF grade B developed (18); the postoperative stay was prolonged (23 days) and complicated with pleural effusion requiring drainage and ascites infection. The diuretic requirement disappeared during follow up. She unfortunately died of recurrent disease at 17 months.

### Case 3

**Figure 4:**
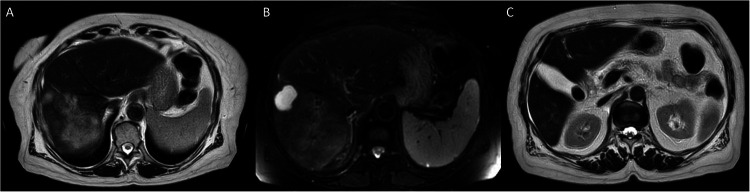
**(A–C)** Heterogeneous mass lesion in the right liver lobe, hyperintense on T2-weighted sequences.

A 69-year-old, female patient with chronic hepatitis B infection controlled with entecavir treatment was diagnosed with a 120 × 100 mm intrahepatic cholangiocarcinoma located in segments 6, 7 and 8 of the liver. She had received four cycles of neoadjuvant chemotherapy and two sessions of transarterial radioembolization in addition to radiotherapy at other institutions. The tumor showed regression and the patient was referred for hepatectomy. She was admitted to hospital two months after the last neoadjuvant chemotherapy session. The right lobe had become atrophic and was mostly occupied by the tumor; the calculated future remnant liver volume was 62% (16) (Figures 4A–C). The platelet counts had varied between 103 and 484 × 10^3^/µL before, but decreased to 90 × 10^3^/µL upon admission. During preoperative cardiac evaluation, this count further declined to 58 × 10^3^/µL. There was no splenomegaly on imaging studies and there were no esophageal varices on upper gastrointestinal endoscopy. Progressive thrombocytopenia could not be readily attributed to previous chemotherapy or portal hypertension. A dexamethasone trial was administered for a possible tentative diagnosis of immune thrombocytopenic purpura; however, the platelet count remained refractory. Hematological malignancies and tumor metastasis were ruled out by bone marrow biopsy. Selective coil embolization of the distal branches of the splenic artery, as well as plugging of the main trunk, were performed empirically, resulting in a gradually increase in platelet count to 159 × 10^3^/µL in 10 days. Subsequently, the patient underwent successful right hepatectomy (H5678) (17), cholecystectomy and cysticostomy.

The maximum level of INR was 1.2 and total bilirubin was 0.5 mg/dL postoperatively. PHLF was not observed (18). Grade-B bile leakage (19) was managed by removing abdominal drains gradually; this resulted in prolonged hospitalization at the patient's request and she was discharged on the 14th postoperative day. On histopathological examination, the nontumoral liver parenchyma exhibited portal-central fibrosis with a stage of 4/6, possibly due to hepatitis B and exaggerated side effects of chemotherapy in a diseased liver. The patient has been followed without recurrence for 21 months.

## Discussion

The experience communicated in this paper is a contribution to the slowly accumulating but favorable literature on the surgical treatment of cholangiocarcinoma and liver metastases in patients with evident portal hypertension (5, 8, 9). Since one of the established options for hepatocellular carcinoma in a similar setting—liver transplant (6)—was not a standard indication for these 3 patients, the alternative method—portal flow modulation—was taken into consideration (3, 5). Splenectomy and splenic artery ligation have been used before for this purpose (3, 5, 7–9). At our center, splenic artery embolization (SAE) was preferred for the following reasons:

1. Splenectomy is an additional procedure that may result in excessive bleeding in a portal hypertensive patient. The purpose of preoperative SAE is to proactively reduce the potential blood loss and to shorten the overall duration of the surgery (15). Also, splenectomy carries risk of splenic-portal vein thrombosis with reported rates between approximately 10% and 25% (7, 20).
2. Performing SAE preoperatively—unlike other portal flow modulation techniques applied during surgery—allows for the preoperative resolution or treatment of potential procedure-related complications, such as splenic infarction or abscess, splenic-portal vein thrombosis, pleural effusion, ascites and prolonged fever (10, 21–23).
3. The effect becomes evident within 1–2 weeks (14, 22, 23) meaning that the surgery does not need to be postponed for a long period.

Portal pressure can not be measured easily before and after SAE because hepatic vein catheterization is required. Non-invasive assessment of portal hypertension, including Doppler ultrasonography and elastography-based methods, may provide indirect information on portal hemodynamics through parameters such as portal vein flow velocity and flow direction; however, these techniques cannot reliably quantify portal pressure and have been mainly validated in patients with chronic liver disease and cirrhosis (24). Because their performance in non-cirrhotic patients with milder disease stages remains limited, we had to use splenomegaly, thrombocytopenia and presence of varices for decision-making. In our small series of 3 patients, all of whom had preoperative thrombocytopenia (platelet counts <100 × 10^3^/µL) (5) we observed a gradual increase in platelet levels by approximately the second week following SAE, reaching values between 159 and 419 × 10^3^/µL, thereby allowing for safe major hepatectomy.

An accumulating body of evidence indicates that in patients with colorectal liver metastases, a significant increase in splenic volume following neoadjuvant chemotherapy is associated with chemotherapy-induced hepatotoxicity, preoperative thrombocytopenia and an increased risk of major postoperative complications, including PHLF (4, 25). Moreover, splenic enlargement, reflecting chemotherapy-related liver injury, has also been linked to impaired liver regeneration following hepatic resection (4). Although advances in chemotherapy have markedly improved outcomes in colorectal liver metastases, prolonged exposure—particularly to oxaliplatin—has been associated with portal hypertension in non-cirrhotic patients (8). Chemotherapy-related sinusoidal injury may increase intrahepatic vascular resistance and lead to clinically relevant portal hypertension despite preserved liver function (8, 25). This was unfortunately observed very clearly in case 1 who developed overt splenic enlargement and thrombocytopenia in the course of chemotherapy. In case 3, chemotherapy probably played a determining role on the background of chronic hepatitis B infection and hepatosteatosis. Although radiotherapy and, transarterial radioembolization, may cause hepatic injury, their predominantly locoregional effect makes a direct contribution to portal hypertension unlikely. In patients with portal hypertension, identified by splenomegaly, thrombocytopenia, and esophageal varices, SAE resulted in improvement of thrombocytopenia and regression of esophageal varices in our patients. Although an increase in spleen volume secondary to preoperative chemotherapy has been associated with thrombocytopenia, changes in spleen volume do not always correlate with changes in platelet counts (25). Incidentally, the third patient exhibited unexplained thrombocytopenia despite the absence of splenomegaly and still benefitted from SAE.

Several groups have reported the complications generally observed after SAE, including a significant incidence of splenic infarction, abscess formation and splenic/portal vein thrombosis (10, 21). To reduce pleural complications such as effusion and pleurisy, limiting embolization to the lower and mid portions of the spleen, preserving the arterial branch(es) to the upper pole are recommended. Intra-procedural angiography helps estimate the devascularized volume, which is ideally maintained between 50% and 70%. Embolizing less than 50% of the spleen often results in suboptimal clinical outcomes, while exceeding 70% increases the risk of complications such as splenic vein thrombosis and abscess formation (10, 14, 23). The published experience suggests that the incidence of the serious complications is significantly lower with using coils and plugs than with embolic agents (26). Although portal pressure reduction may be more efficient and rapid with plugs than with coils (27), in two other patients who underwent SAE for other reasons, late collateral development to the arterial segment distal to the vascular plug was observed and secondary interventions were required (unpublished observation). Therefore, we prefer using coils as distally as possible (without embolic material) to avoid thrombus formation at the capillary levels and simultaneously decrease the risk of arterial recanalization beyond the proximally placed coils (21, 22). The marked increase in thrombocytopenia has been associated with the area of splenic infarction, and it has been emphasized that large infarct area can be achieved in a controlled fashion with distal splenic artery embolization (21, 22). In the two patients with splenomegaly our series, we opted for selective distal embolization affecting 50%–70% of the spleen, which led to the evident resolution of thrombocytopenia without complication. The third patient did not have splenomegaly; coil embolization of the distal branches of the splenic artery, as well as plugging of the main trunk, were performed empirically.

Limitations of our study are its retrospective nature, small number of cases and unavailability of portal pressure measurements before or after splenic artery embolization. In other words, the effect of SAE on portal pressure reduction has not been directly determined. It should also be noted that the right lobe volumes of the patients (53%, 37%, 38% respectively) who underwent major hepatectomy were less than 60%. Therefore, the success of SAE in more extensive hepatectomies can not be predicted.

SAE is considered to be the radiological counterpart of SAL for portal flow modulation. SAL has been performed intraoperatively during hepatectomy or liver transplantation to alleviate portal pressure (5, 9, 15). SAE, on the other hand, has mainly been utilized for the prevention and the treatment of small-for-size syndrome in transplantation (11, 15). Although previous reports have described portal flow modulation strategies in patients with portal hypertension undergoing hepatic surgery, published experience with preoperative SAE for this purpose remains extremely limited. Our case series adds to the limited published experience (six previous cases) on the prophylactic use of SAE before hepatectomy to modulate portal hemodynamics and prevent postoperative complications (28, 29).

In conclusion, SAE demonstrates efficacy and safety in managing chemotherapy-, biliary obstruction- and viral hepatitis- induced liver damage that manifests with portal hypertension. The platelet count serves as a useful surrogate marker for monitoring the outcomes of SAE. Subtle signs of portal hypertension may reflect increased the risk of PHLF even in patients without liver dysfunction (5). In this context, preoperative SAE emerges as a safe and effective portal pressure modulation method before major hepatectomy.

## Data Availability

The original contributions presented in the study are included in the article/Supplementary Material, further inquiries can be directed to the corresponding authors.
